# Mixed Fermentation Using *Saccharomyces cerevisiae* var. *boulardii* and Kombucha Culture as a Strategy for the Production of Mead with Antimicrobial Activity

**DOI:** 10.3390/foods15162863

**Published:** 2026-08-17

**Authors:** Ricardo Donizete Teixeira, Handray Fernandes de Souza, Ana Catarina Costa, Felipe Donizete Teixeira, Rafael Lopes de Alcântara, Igor Viana Brandi, Eliana Setsuko Kamimura, Catarina Prista

**Affiliations:** 1 Department of Food Engineering, School of Animal Science and Food Engineering, Universidade de São Paulo, Av. Duque de Caxias Norte, 225, Pirassununga 13635-900, São Paulo, Brazil; felipeteixeira1@usp.br (F.D.T.); rafaellopesdealcantara@usp.br (R.L.d.A.); elianask@usp.br (E.S.K.); 2 LEAF—Linking Landscape, Environment, Agriculture and Food Research Center, Associated Laboratory TERRA, Instituto Superior de Agronomia, University of Lisbon, Tapada da Ajuda, 1349-017 Lisbon, Portugal; acatarinacosta@isa.ulisboa.pt (A.C.C.); cprista@gmail.com (C.P.); 3 Institute of Agricultural Sciences, Universidade Federal de Minas Gerais, Av. Universitária, 1000, Montes Claros 39404-547, Minas Gerais, Brazil; ibrandi@hotmail.com

**Keywords:** eucalyptus honey, rosemary honey, alcoholic beverage, fermentation, antimicrobial effect, pathogenic bacteria, spoilage yeasts

## Abstract

This study evaluated the antimicrobial activity of meads produced by mixed fermentation of *Saccharomyces cerevisiae* var. *boulardii* and a kombucha culture, using eucalyptus- and rosemary-monofloral honeys. Antimicrobial activity was investigated in vitro against pathogenic bacteria (*Escherichia coli*, *Salmonella enterica*, *Listeria innocua*, *Staphylococcus aureus*, and *Bacillus cereus*) and spoilage yeasts, over 28 days of storage. All tested matrices were autoclaved at 121 °C for 15 min before antimicrobial evaluation to eliminate viable fermentation microorganisms. Additionally, assays were conducted in simulated food systems at different substrate concentrations, characterizing organic acids, reducing sugars, and volatile compounds. Unfermented musts allowed survival of *L. innocua* and *B. cereus*, with rosemary honey showing stronger antimicrobial effects than eucalyptus. Kombucha exerted strong initial inhibition, possibly associated with its high organic acid concentrations, but did not completely inhibit the growth of Gram-positive bacteria. No pathogenic bacterial growth was detected in the fermented mead matrices, possibly due to reduced fermentable sugars, ethanol, organic acids, and other fermentation metabolites. Spoilage yeasts showed high tolerance to acid and osmotic stress, and efficacy decreased in substrate-rich systems. Importantly, the antimicrobial activity reported here refers specifically to autoclaved fermented matrices and does not necessarily apply to untreated fermented beverages.

## 1. Introduction

Antimicrobial activity is broadly defined as the ability of a substance or agent to inhibit microorganisms, including bacteria, viruses, fungi, and protozoa. In foods, antimicrobial activity is particularly relevant for controlling pathogenic and spoilage microorganisms, thereby contributing to food safety, preservation, and shelf-life extension [1,2,3].

Among natural antimicrobials, compounds of animal, plant, and microbial origin have been widely investigated, including enzymes, bioactive peptides, phenolic compounds, organic acids, and metabolites produced during fermentation processes [4,5]. Honey is a sugar-rich natural product mainly composed of fructose and glucose, together with smaller amounts of proteins, enzymes, amino acids, minerals, vitamins, organic acids, and phenolic compounds [6]. Its antimicrobial activity has been associated with the combined effects of low water activity, high osmolarity, low pH, hydrogen peroxide production, and bioactive compounds such as phenolic acids, flavonoids, and defensin-1 [7,8,9]. These characteristics have been associated with antimicrobial effects against different Gram-positive and Gram-negative bacteria and yeasts, as reported for eucalyptus honey and other honey varieties [6,10].

In parallel, fermented beverages have attracted increasing interest due to their functional properties and the compounds formed during fermentation, including organic acids, ethanol, carbon dioxide, modified phenolic compounds, and other secondary metabolites that may contribute to antimicrobial activity [11]. Among these beverages, kombucha stands out as a fermented black and/or green tea (*Camellia sinensis*) beverage generally produced using a symbiotic culture of acetic acid bacteria and yeasts, known as a “symbiotic culture of bacteria and yeast” (SCOBY) [12]. Its antimicrobial activity has been associated mainly with low pH, organic acids, particularly acetic acid, and phenolic compounds generated or modified during fermentation. However, these characteristics depend on several processing conditions, including tea and sugar concentrations, tea type, temperature, fermentation time, and the microbial composition of the starter culture [13,14].

Other microorganisms have also been investigated for their potential antimicrobial properties, including the probiotic yeast Saccharomyces cerevisiae var. boulardii (*S. boulardii*), which exhibits physiological characteristics that favor its activity under gastrointestinal conditions and has been associated with antimicrobial effects against several pathogenic microorganisms [11]. Its effects have been related to mechanisms such as the production of antimicrobial factors capable of interfering with microbial toxins and the interaction of cell-wall mannose residues with pathogenic microorganisms and their components [15,16]. Some of these cell-associated properties may remain after yeast inactivation, supporting the investigation of S. boulardii-derived preparations as potential postbiotics [17]. Thus, these effects should be considered according to the physiological state of the yeast, since viable cells may exert probiotic effects, whereas nonviable cells may retain specific cell-associated properties.

Consumer interest in healthier, minimally processed foods and products with reduced use of chemical preservatives has increased the demand for “clean label” alternatives [18,19,20] and functional foods capable of providing benefits beyond basic nutrition [21,22]. This trend has stimulated the development of probiotic beverages [23,24,25,26] and has also expanded to less explored sectors, including alcoholic beverages [27]. In this context, potentially probiotic mead produced through mixed fermentation using *S. boulardii* and kombucha microorganisms represents an innovative approach [28,29]. However, the development of such products requires strategies to ensure microbiological safety and stability while limiting the use of conventional chemical preservatives [30]. The combination of naturally bioactive raw materials and selected fermentation microorganisms may therefore represent an alternative strategy for controlling pathogenic and spoilage microorganisms and expanding the potential application of fermentation-derived products in food preservation.

In view of the above, this study aimed to evaluate the antimicrobial activity associated with soluble fermentation-derived compounds of mead produced by mixed fermentation of *Saccharomyces cerevisiae* var. *boulardii* and a kombucha culture against foodborne pathogens and contaminating yeasts, and its potential application as a food preservative ingredient. In addition to the intrinsic antimicrobial activity of the beverage, its application potential was investigated through simulations under different nutrient availability levels, aiming to mimic real conditions for the use of mead as a natural preservative.

## 2. Materials and Methods

### 2.1. Materials

*Saccharomyces cerevisiae* var. *boulardii* (ISA 1028) was obtained from the yeast culture collection of the Instituto Superior de Agronomia (ISA, University of Lisbon, Lisbon, Portugal). The symbiotic culture of bacteria and yeasts (SCOBY) used for kombucha production was obtained from the microbial culture collection of the Laboratory of Microbial Bioenergetics (LBM) at ISA (Lisbon, Portugal). Eucalyptus honey (Euromel, Penamacor, Portugal) predominant floral origin of *Eucalyptus* spp.; rosemary honey (Euromel, Penamacor, Portugal) predominant floral origin of *Lavandula* spp.; and green tea (Lipton^®^ Green Tea, *Camellia sinensis* leaves) were purchased from a local supermarket (Lidl, Lisboa, Portugal).

### 2.2. Methods

#### 2.2.1. Kombucha and Mead Production

Kombucha and mead were produced based on the method described by [28] with adaptations. The average physicochemical parameters and the stability of these systems have already been previously characterized and reported in the literature [28,29].

For kombucha preparation, commercial green tea bags (*Camellia sinensis*; Lipton, Classic) were used at a concentration of 6 g/L and infused in potable water heated to 80 °C for 10 min. After infusion, the liquid was supplemented with 50 g/L of commercial granulated sugar. The sweetened tea was cooled to 25 °C and inoculated with 10% (*w*/*v*) SCOBY and 10% (*v*/*v*) previously fermented kombucha, used as a starter culture. The kombucha was fermented at 25 °C for 20 days. The physicochemical and microbiological evolution of kombucha produced using the same formulation and processing conditions was previously characterized by [28].

The mead was prepared in graduated neutral-glass reagent bottles, with 3/5 of the total volume filled with must, allowing sufficient headspace for CO_2_ release during fermentation. The amounts of honey and potable water were calculated in advance to obtain a must with an initial soluble solids content of 25 °Brix. The water was heated to 65 °C, and the honey was dissolved while maintaining this temperature. Subsequently, the mixture was pasteurized at 65 °C for 30 min and cooled to 25 °C before inoculation with the microbial cultures.

After cooling, 0.75 g/L of *Saccharomyces cerevisiae* var. *boulardii* (ISA 1028) and 25 mL/L of kombucha were added. The fermentation vessels were sealed with lids equipped with airlock systems, ensuring anaerobic conditions. Fermentation was carried out for 9 days at 25 °C in a BOD incubator. Two types of mead were produced, differentiated by the floral origin of the honey used: mead made with rosemary honey (RM) and mead made with eucalyptus honey (EM).

#### 2.2.2. In Vitro Antimicrobial Activity of the Products

To evaluate the in vitro antimicrobial activity, different samples were analyzed: eucalyptus honey must without microorganism inoculation (EHM), rosemary honey must without microorganism inoculation (RHM), kombucha (K), eucalyptus honey must immediately after microorganism inoculation (EM-T0), rosemary honey must immediately after microorganism inoculation (RM-T0), fermented eucalyptus mead (EM), and fermented rosemary mead (RM). However, to eliminate viable microorganisms originating from the fermentation cultures and prevent their proliferation during the antimicrobial challenge assays, all the tested samples were sterilized in a vertical autoclave (model MLS-3020U, Panasonic, Osaka, Japan) at 121 °C for 15 min. This procedure prevented the growth of these microorganisms in the culture medium, and minimized potential interference with the development of the contaminating bacteria and yeasts used in the evaluation of antimicrobial activity.

The in vitro antimicrobial activity assay was performed according to the spot-on-lawn method [31,32,33], with adaptations. Table 1 presents the pathogenic and contaminating microorganisms tested, obtained from the bacterial and yeast collections of the Instituto Superior de Agronomia (ISA), University of Lisbon, Portugal.

In general, typical contaminating yeast strains were cultivated in Yeast Extract Peptone Dextrose Agar medium (YPD; Merck, Darmstadt, Germany) at 25 °C, while foodborne pathogenic bacteria were grown in Brain Heart Infusion medium (BHI; Merck, Darmstadt, Germany) at 37 °C until reaching the mid-exponential growth phase.

Subsequently, an appropriate volume of each microbial suspension was inoculated into screw-cap cryogenic microtubes (1.2 mL) containing 1 mL of the respective tested sample (EHM, RHM, K, EM-T0, RM-T0, EM, or RM), resulting in a final concentration of 10^5^ CFU/µL in each tube. Two replicate microtubes were prepared for each microorganism–sample combination and maintained at 23 °C. Decimal serial dilutions were performed from 10^5^ CFU/µL to 10^0^ CFU/µL in 96-well microplates. Afterwards, 3 µL aliquots of each dilution were transferred onto Petri dishes containing the appropriate culture medium, BHI for bacteria and YPD for yeasts, using a pin replicator. After inoculation, plates containing contaminating yeasts were incubated in a BOD incubator at 25 °C, whereas plates containing pathogenic bacteria were incubated at 37 °C. No detectable growth was defined as the absence of visible microbial growth at the evaluated inoculum concentration after 72 h of incubation. The in vitro antimicrobial activity assay was performed at 0, 7, 14, and 28 days, in triplicate, using samples stored in cryogenic microtubes. EHM and RHM were included as unfermented and uninoculated comparison matrices, whereas EM-T0 and RM-T0 represented the corresponding musts immediately after inoculation with the fermentation cultures. These matrices were included to compare the effects of culture inoculation and fermentation. However, no independent positive growth control consisting of the challenge microorganism incubated under the same conditions in the absence of the tested matrices was included in this assay.

#### 2.2.3. Antimicrobial Activity in Systems with Different Nutrient Availability

In a second methodological approach, the aim was to simulate the application of fermented beverages in food systems with varying nutrient availability. For this purpose, experimental formulations were prepared by combining three different basal media: a 4% agar solution, used to simulate systems with low nutrient availability, and Yeast Extract Peptone Dextrose (YPD) and Brain Heart Infusion (BHI), used to simulate nutrient-rich systems.

The basal media were used at a fixed proportion of 50% (*v*/*v*), with the autoclaved tested samples (EHM, RHM, K, EM-T0, RM-T0, EM, and RM) incorporated at concentrations of 10%, 25%, and 50% (*v*/*v*). The final volume was completed with sterile distilled water up to 100%, resulting in water proportions of 40%, 25%, or no water, respectively, as shown in Table 2.

In total, 72 experimental formulations were obtained (3 basal media × 3 sample concentrations × 7 tested samples), in addition to three control conditions consisting of 100% YPD, 100% BHI, and 100% agar medium.

The microorganisms (Table 1) were inoculated into microtubes containing the specific medium at a concentration of 10^8^ CFU/mL and subsequently diluted in 96-well microplates within the range of 10^5^ to 10^0^ CFU/µL. Aliquots of 3 µL from each dilution were transferred onto Petri dishes containing the experimental media (Table 2). Antimicrobial activity was evaluated after 72 h of incubation, and all assays were conducted in independent triplicates, ensuring reproducibility and reliability of the results.

#### 2.2.4. Characterization of Organic Acids, Reducing Sugars, and Volatile Compounds Profile by High-Performance Liquid Chromatography (HPLC)

The determination of organic acids, reducing sugars, and volatile compounds was performed using high-performance liquid chromatography (HPLC), according to the method described by [34], with modifications. The adaptations mainly refer to sample preparation. Aliquots for HPLC analysis were collected after the sterilization treatment described in Section 2.2.2 (121 °C for 15 min), so the chemical characterization reflects the composition of the same sterilized matrices used in the antimicrobial activity assays. 

In general, the samples were previously homogenized and degassed to remove dissolved carbon dioxide. Subsequently, they were centrifuged at 3150× *g* for 10 min at 4 °C to separate suspended particles and residual biomass, and the supernatant was carefully collected. The obtained supernatant was diluted 1:10 with 50 mM H_2_SO_4_, centrifuged 10 min at 16,000× *g* and the supernatant was then diluted 1:2 in the same acid concentration and filtered through a 0.22 µm membrane. When necessary, the filtered supernatant was diluted in ultrapure water to fit within the linear calibration range of the method.

The analyses were carried out using a Chromaster HPLC system (Hitachi, Tokyo, Japan), equipped with a Rezex™ ROA Organic Acid H^+^ (8%) column (Phenomenex, Torrance, CA, USA), maintained at 65 °C. The mobile phase consisted of 5 mM H_2_SO_4_, with a flow rate of 0.5 mL/min. Organic acids were detected using a UV-Vis detector (5420), while sugars and ethanol and glycerol were quantified using a refractive index detector (5450). The injection volume was 20 µL, and each sample was analyzed in independent triplicate. Quantification was performed using analytical curves obtained from external standards prepared under the same chromatographic conditions.

#### 2.2.5. Statistical Analysis

To compare the concentrations of organic acids, reducing sugars, and volatile compounds among the different samples, R software (version 4.4.2; R Foundation for Statistical Computing, Vienna, Austria) was used. Initially, the data were evaluated considering a Completely Randomized Design (CRD). However, the assumptions required for the application of parametric tests were violated. Residual normality was assessed using the Shapiro–Wilk test, while variance homogeneity was verified using a homogeneity test. The results indicated that the residuals did not follow a normal distribution and that variances could not be considered homogeneous (*p* < 0.05).

Therefore, a nonparametric approach was used, with the Kruskal–Wallis test for global comparisons among groups, at a significance level of 5% (*p* < 0.05). When significant differences were detected, post hoc analysis was performed using Conover’s test, with *p*-value adjustment by the Holm method, to identify pairwise differences among samples from the same floral origin.

## 3. Results and Discussion

### 3.1. In Vitro Inhibitory Capacity of the Products Against Pathogenic Bacteria

**Figure 1:**
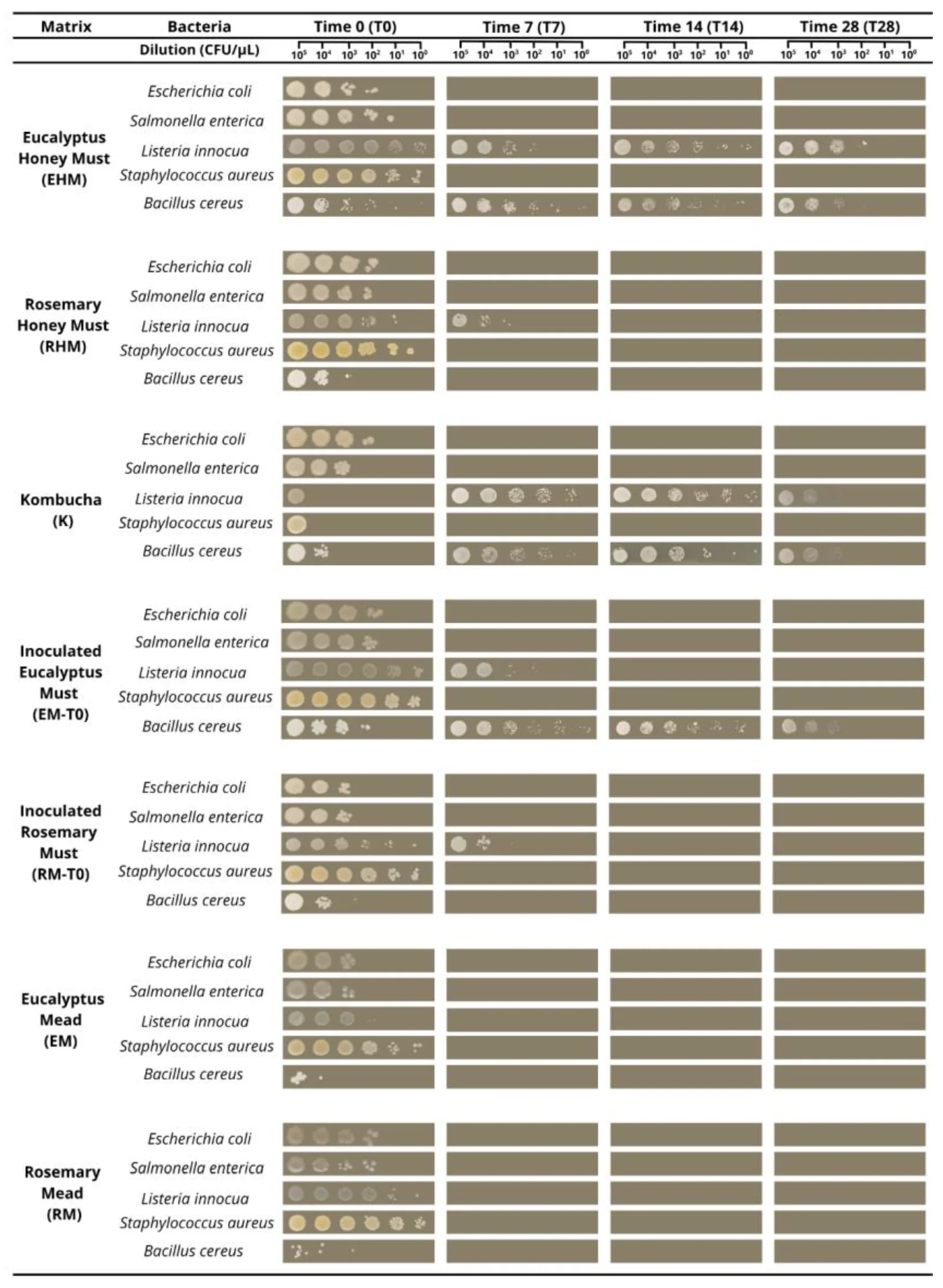
Evaluation of in vitro antimicrobial activity against pathogenic bacteria over 28 days.

Figure 1 presents the in vitro evaluation of the antimicrobial activity of eucalyptus honey must without microorganism inoculation (EHM), rosemary honey must without microorganism inoculation (RHM), kombucha (K), eucalyptus honey must immediately after microorganism inoculation (EM-T0), rosemary honey must immediately after microorganism inoculation (RM-T0), fermented eucalyptus mead (EM), and fermented rosemary mead (RM) against *Escherichia coli*, *Salmonella enterica*, *Listeria innocua*, *Staphylococcus aureus*, and *Bacillus cereus*.

It should be highlighted that both kombucha and honey present antimicrobial mechanisms that are widely described in the literature. During fermentation, kombucha accumulates organic acids, such as acetic, lactic, gluconic, and glucuronic acids, as well as polyphenols, compounds that contribute to pH reduction and the inhibition of enteric bacteria [35,36]. In turn, honey exerts antimicrobial activity due to the combination of different factors, including the high osmotic pressure resulting from its elevated sugar content, low pH, hydrogen peroxide production through the action of glucose oxidase, and the presence of phenolic compounds [37] and flavonoids. Furthermore, honey composition varies according to its floral origin; metabolomic studies have identified L-phenylalanine and raffinose as markers of rosemary honey, and Amadori compounds as markers of eucalyptus honey [38].

As presented in Figure 1, the growth of *L. innocua* and *B. cereus* in EHM persisted throughout the 28 days of storage. However, *B. cereus* growth was observed only at the highest inoculation concentration (10^3^ CFU/µL), whereas *L. innocua* showed fluctuating behavior during the evaluation period, with growth at 10^2^ CFU/µL after 28 days. In contrast, in RHM, *L. innocua* was detected only after 7 days at a dilution of 10^3^ CFU/µL and was no longer observed thereafter. This difference between EHM and RHM confirms that the chemical profile of honey directly influences the antimicrobial activity of the matrices. Rosemary honey presents a phenolic composition distinct from that of eucalyptus honey [38], as well as a greater diversity of organic acids in its composition, as shown in Table 3. Therefore, the matrix prepared with rosemary honey demonstrated greater antimicrobial activity than the matrix prepared with eucalyptus honey.

According to Figure 1, the isolated kombucha (K) exhibited high inhibitory activity at time zero, particularly against *L. innocua*, *S. aureus*, and *B. cereus*. This effect may be associated with its high acidity, characterized by the presence of acetic acid (0.250 g/100 g), lactic acid (0.156 g/100 g), and malic acid (0.243 g/100 g), all at concentrations higher than those observed in the other samples (Table 3). However, during the 28 days of storage, growth of *L. innocua* (10^4^ CFU/µL) and *B. cereus* (10^3^ CFU/µL) was observed, indicating that, despite the high acidity of the matrix, the stress imposed by the compounds present was not sufficient to completely inhibit the growth of these Gram-positive bacteria. As described in the literature, *Bacillus cereus* is an opportunistic human pathogen capable of causing diarrheal and emetic syndromes through the production of enterotoxins and cereulide. This bacterium is widely distributed in the environment, especially in soil, and possesses a peptidoglycan-rich cell wall and the ability to form highly resistant spores. These spores can survive in alcoholic and fermented beverages for extended periods, remaining viable for weeks and subsequently germinating under favorable conditions [39,40]. It can also be inferred that the acidic pH of kombucha [25,41] may have contributed to the inactivation of part of the vegetative cells of *B. cereus*, thereby possibly favoring sporulation. Although spore formation was not directly evaluated in the present study, surviving spores could have subsequently germinated when transferred to a suitable nutrient medium. However, a gradual reduction in growth was observed over the 28-day period, with *B. cereus* growth occurring only at the highest inoculum concentrations during the final storage periods [25,41]. On the other hand, the persistence of *L. innocua* in kombucha (K) and in the eucalyptus honey must without microbial inoculation (EHM) (Figure 1) may be associated with acid-stress tolerance, as specific adaptation mechanisms have been reported in some *Listeria* species [42,43]. For example, the glutamate decarboxylase (GAD) system converts glutamate into γ-aminobutyric acid (GABA) and exports GABA in exchange for glutamate, consuming protons and stabilizing intracellular pH. This system involves membrane-localized antiporters and decarboxylases. In addition, the sigma factor B (SigB) regulates stress-response genes and is involved in the activation of the GAD system and other survival pathways [42,43]. Thus, the survival of *L. innocua* in the musts reflects its intrinsic capacity for physiological adaptation.

In the eucalyptus and rosemary honey musts inoculated with the fermentation microorganisms (EM-T0 and RM-T0, respectively), behavior similar to that observed in the respective uninoculated controls (EHM and RHM) was observed, as shown in Figure 1. In EM-T0, *Listeria innocua* exhibited growth up to the 7th day of storage at the dilution of 10^4^ CFU/µL, whereas *Bacillus cereus* remained viable throughout the entire evaluation period at the concentration of 10^3^ CFU/µL for up to 28 days. In contrast, in RM-T0, only *L. innocua* growth was observed up to the 7th day at the concentration of 10^4^ CFU/µL, with no growth of any other microorganism detected after this period. These results suggest that the immediate addition of *Saccharomyces boulardii* and the kombucha culture to the honey must did not substantially increase antimicrobial activity compared with the uninoculated musts. This interpretation is supported by the organic acid profile (Table 3), as in EM-T0 only formic and lactic acids were detected at additional concentrations compared with EHM, where these compounds were not identified. In contrast, RM-T0 exhibited an organic acid profile similar to that observed in RHM. Taken together, these results indicate that the short interaction period between the inoculated microorganisms and the substrate may not have been sufficient to produce antimicrobial inhibitory effect against the evaluated microorganisms.

Regarding the eucalyptus (EM) and rosemary (RM) meads, no bacterial growth was observed during the 28-day storage period, as shown in Figure 1. This behavior may be associated with physicochemical changes occurring during fermentation and subsequent heat treatment. However, because the complete matrices were evaluated, the contribution of individual compounds or compound families to the antimicrobial activity could not be independently determined. During fermentation, there was substantial consumption of fermentable sugars, especially glucose and fructose (Table 3), resulting in ethanol production and modification of the medium conditions. During mixed alcoholic fermentation, the inoculated *S. boulardii* strain, in association with microorganisms derived from the kombucha culture, primarily converted glucose and fructose into ethanol and carbon dioxide. Statistically significant reductions in fructose concentration were observed, from 11.996 to 10.007 g/100 g between EM-T0 and EM, and from 12.914 to 12.464 g/100 g between RM-T0 and RM. Similarly, glucose concentration decreased from 8.345 to 4.937 g/100 g between EM-T0 and EM, and from 8.364 to 7.018 g/100 g between RM-T0 and RM. These results indicate effective consumption of the available fermentable sugars, although to different extents in the evaluated systems. However, part of the carbon flux may also have been directed toward secondary metabolic pathways associated with organic acid formation, thereby contributing to the final acid profile presented in Table 3. In addition, microorganisms introduced through the kombucha culture may have promoted further acidification through the conversion of residual sugars and ethanol into organic acids, as previously observed in [28].

Although residual ethanol contents of 1.201% and 0.481% were detected in EM and RM, respectively, these relatively low concentrations are unlikely to explain the observed bacterial inhibition on their own. Nevertheless, residual ethanol may have contributed to the combined antimicrobial effect by affecting bacterial membrane fluidity and permeability and facilitating the action of other inhibitory compounds present in the matrices [44]. Therefore, the absence of detectable growth of pathogenic microorganisms under the conditions tested should not be attributed to a single factor, but rather to the synergistic action of multiple hurdles, including the reduced availability of fermentable nutrients, the residual presence of ethanol, medium acidification, and the possible production of bioactive metabolites resulting from the metabolic activity of *Saccharomyces boulardii* and the microorganisms present in the kombucha culture. This set of factors is consistent with the concept of hurdle technology, according to which the combination of different sublethal stresses acts synergistically to prevent the survival and development of spoilage and pathogenic microorganisms [15].

Considering the overall results, the comparison of the heat-treated unfermented and fermented matrices indicates that fermentation-related changes were associated with the stronger antibacterial activity observed in the meads. Nevertheless, the present experimental design does not allow this activity to be attributed to a specific compound or family of compounds. The inhibition probably resulted from the combined influence of several physicochemical and chemical factors, whose individual contributions require further investigation. Although honey and kombucha exhibit antimicrobial effects inherent to their compositions, only the metabolic modifications induced by fermentation were sufficient to generate conditions that simultaneously inhibited Gram-positive and Gram-negative bacteria throughout the entire storage period. Collectively, these results point to the central role of fermentation in enhancing the microbiological stability and biopreservative potential of the evaluated products. Nevertheless, because all matrices were autoclaved before testing, fermentation cannot be established as the sole determinant of the observed activity, as thermal modifications may have also contributed to the results. Therefore, the antimicrobial activity reported in this study refers specifically to heat-treated fermented matrices and does not necessarily apply to untreated fermented beverages.

### 3.2. In Vitro Inhibitory Capacity of the Products Against Contaminating Yeasts

**Figure 2:**
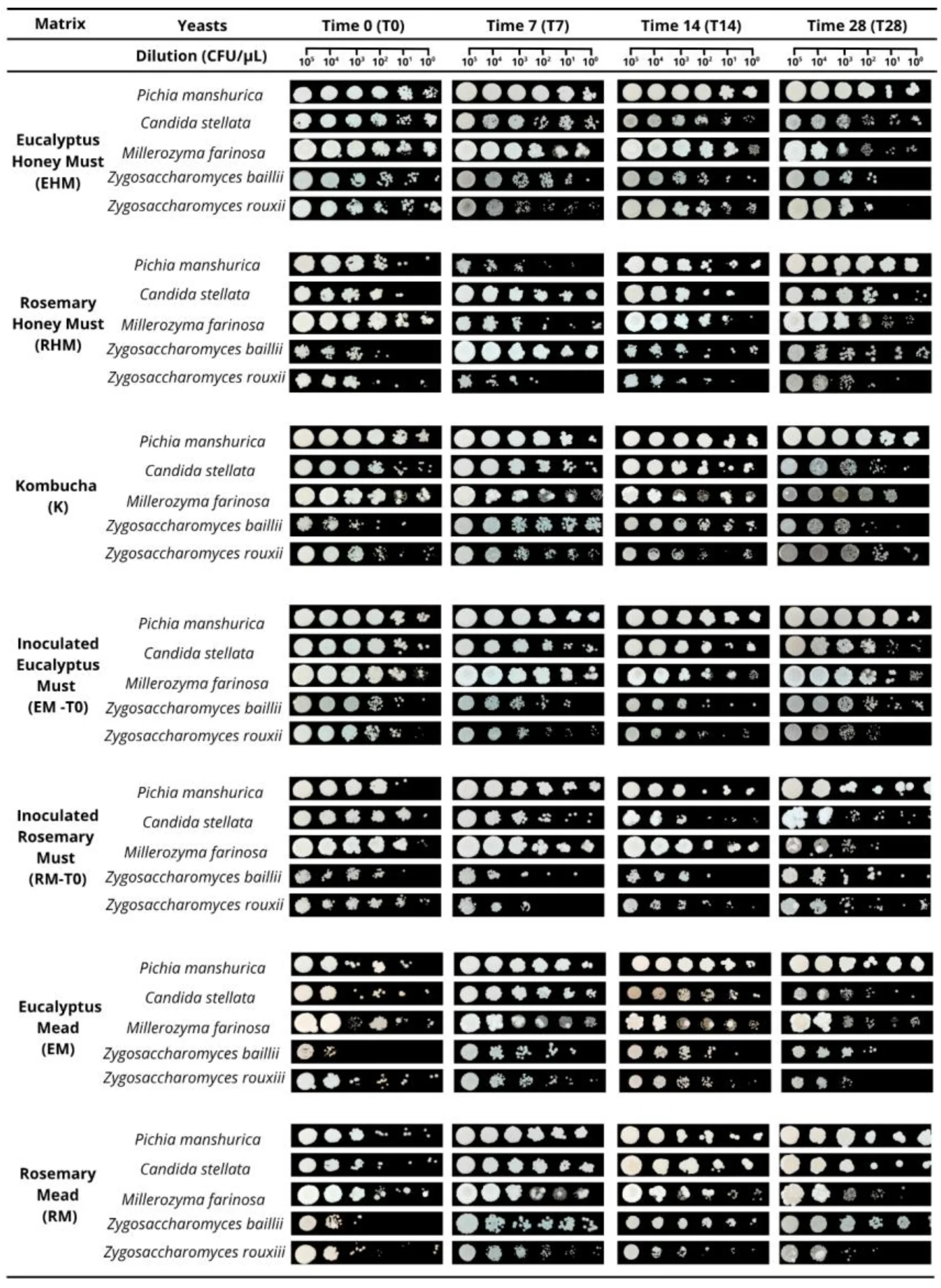
Evaluation of the in vitro antimicrobial activity against contaminating yeasts over 28 days.

Regarding the antimicrobial activity against the contaminating yeasts *Pichia manshurica*, *Candida stellata*, *Millerozyma farinosa*, *Zygosaccharomyces bailii*, and *Zygosaccharomyces rouxii*, no reduction in the microbial population was observed during the 28 days of storage, as shown in Figure 2. This behavior is consistent with the high adaptive capacity of these species, which are recognized for their tolerance to environments with high osmotic pressure, low pH values, the presence of organic acids, and moderate ethanol concentrations, conditions similar to those found in the musts and meads evaluated. Overall, the results indicate that the meads produced from different types of honey exhibited greater antimicrobial efficacy against bacteria (Figure 1) than against contaminating yeasts (Figure 2), highlighting differences in the susceptibility of these microbial groups. In this context, the absence of an inhibitory effect against the evaluated yeasts suggests that the antimicrobial factors present in the samples, including medium acidification, residual ethanol content, and other metabolites produced during fermentation, were insufficient to overcome the physiological resistance and adaptation mechanisms of these species.

Regarding *Pichia manshurica*, its occurrence has been reported in different fermentation processes, including wine and vinegar production, and cocoa bean fermentation. This species exhibits a high capacity to adapt to adverse environments, tolerating low pH values, elevated temperatures, and the presence of ethanol, and it produces pectinolytic enzymes. Furthermore, species of the genus *Pichia* have demonstrated the ability to colonize surfaces and form biofilms at the air–liquid interface, as well as resistance to organic acids, characteristics that favor their survival and persistence in fermented matrices. In industrial environments, these yeasts may also act as recurrent contaminants, colonizing processing surfaces and filtration systems in wineries [41,45]. Collectively, these physiological and ecological characteristics help explain the persistence of *P. manshurica* in the evaluated samples, even under acidified conditions and in the presence of other antimicrobial factors generated during fermentation.

*Candida stellata*, in turn, is frequently reported in grape musts and winemaking processes, particularly during the early stages of alcoholic fermentation [46]. This species exhibits a high degree of adaptation to fermentative environments and can tolerate moderate ethanol concentrations of up to approximately 9% (*v*/*v*), although its proliferation may be associated with deterioration of product quality. Furthermore, studies have investigated its biotechnological potential due to its ability to produce esters and other volatile compounds that contribute to the aromatic and sensory profile of fermented beverages, such as wines and vinegars [47]. Its persistence in these environments is favored by its tolerance to low pH values and its ability to compete efficiently in matrices with high concentrations of fermentable sugars [48]. In this context, the residual ethanol contents observed in the evaluated meads, below 1.5% (*v*/*v*) (Table 3), probably did not constitute a sufficiently severe antimicrobial barrier to compromise the survival and growth of this species.

Regarding *Millerozyma farinosa*, its remarkable ability to tolerate osmotic and saline stress conditions should be highlighted, as it is classified as an osmotolerant and halotolerant yeast [49]. One of the main mechanisms responsible for this adaptation is the intracellular accumulation of compatible solutes, such as glycerol and other polyols, which function in osmotic adjustment and in maintaining cellular integrity in environments with high solute concentrations [49]. Therefore, the high sugar concentrations present in the evaluated matrices may have favored the survival and maintenance of the viability of this species by reducing the effects of osmotic stress and contributing to its persistence throughout the storage period.

Among the evaluated yeasts, *Zygosaccharomyces bailii* and *Zygosaccharomyces rouxii* deserve special attention because of their well-established relevance as spoilage agents in foods and beverages. *Z. bailii* is considered one of the most resistant spoilage yeasts in the food industry, particularly in acidic products, due to its high tolerance to weak organic acids used as preservatives, as well as to ethanol and high sugar concentrations [50]. Additionally, this species exhibits fructophilic behavior [51] preferentially utilizing fructose as a carbon source when available, a characteristic particularly relevant in honey-derived matrices and musts with high sugar content. Therefore, the combination of acid tolerance, osmotolerance, and ethanol resistance likely contributed to its survival in the analyzed samples.

Similarly, *Z. rouxii* exhibits a remarkable capacity to adapt to environments with low water activity, high osmotic pressure, and low pH, being able to grow under extremely acidic conditions, with pH values close to 2.0. Its tolerance to osmotic stress is mainly associated with the intracellular accumulation of glycerol and other compatible solutes, as well as changes in the lipid composition of the cell membrane that promote the maintenance of structural and functional integrity under adverse conditions [52]. Furthermore, its high fermentative capacity may result in the production of carbon dioxide and volatile compounds, changes frequently associated with the spoilage of concentrated and sugar-rich products. Collectively, the physiological characteristics of these species help explain the absence of an inhibitory effect observed throughout storage, highlighting the high ecological and metabolic robustness of these yeasts in fermented environments.

Overall, the evaluated yeasts share physiological adaptation mechanisms that promote their survival in fermentative environments, including osmotic adjustment through the accumulation of compatible solutes, modifications in the composition and functionality of the cell membrane, tolerance to organic acids, and resistance to moderate ethanol concentrations. Although fermentation proved effective in inhibiting bacterial microorganisms, the conditions established in the meads, characterized by moderate acidity and low residual ethanol contents (Table 1), were not sufficiently severe to exceed the tolerance limits of these yeasts. Consequently, these species maintained their viability throughout the storage period, demonstrating their high capacity to adapt to the physicochemical conditions present in the evaluated matrices. Future studies should investigate the optimization of fermentation conditions to reduce residual fermentable sugars and increase the production of antifungal metabolites. These strategies could also be combined with nonthermal preservation technologies, such as high-pressure processing or pulsed electric fields, to improve the control of spoilage yeasts.

### 3.3. In Vitro Antimicrobial Activity of the Products Under the Effect of Different Substrate Concentrations

**Figure 3:**
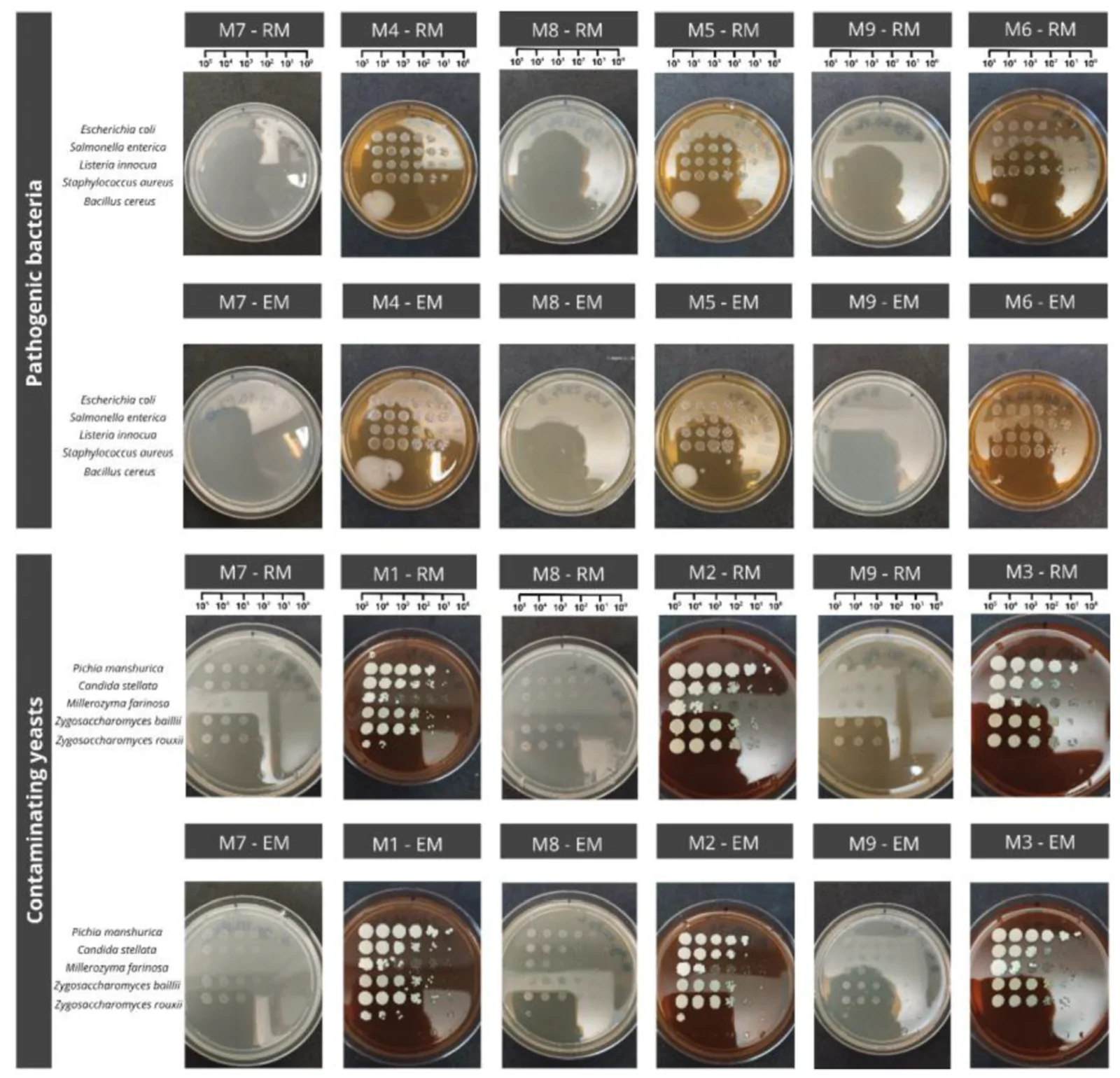
Antimicrobial activity of rosemary (RM) and eucalyptus (EM) meads in experimental systems with different nutrient availabilities. Media M1–M9 correspond to the formulations described in Table 2, consisting of nutrient basal medium or agar supplemented with the samples at different concentrations (10%, 25%, and 50%, *v*/*v*) and sterile water.

The antimicrobial activity was evaluated after incorporating the samples at concentrations of 10%, 25%, and 50% (*v*/*v*) into systems with different nutrient availabilities (Table 2), aiming to investigate the potential application of these matrices as food preservatives. In nutrient-rich culture media, such as YPD and BHI, none of the samples exhibited an inhibitory effect on the growth of pathogenic bacteria or yeasts. These results suggest that the high availability of nutrients favored metabolic recovery and microbial proliferation, reducing the effectiveness of the antimicrobial action exerted by the evaluated matrices. Furthermore, the meads produced with eucalyptus honey (EM) and rosemary honey (RM), which had previously demonstrated antimicrobial activity against pathogenic bacteria, were not able to effectively inhibit microbial growth at the tested concentrations of up to 50% (*v*/*v*) (Figure 3).

In agar medium without nutritional supplementation, no bacterial growth was observed under any of the evaluated conditions. For rosemary (RM) and eucalyptus (EM) meads (Figure 3), this behavior may be attributed to the higher antimicrobial activity of these matrices, associated with the low availability of fermentable sugars and other essential nutrients required for bacterial metabolism. In the case of the musts and kombucha, the absence of bacterial growth was likely related to the nutritional limitation imposed by the experimental system, since the non-supplemented agar medium did not provide sufficient substrates to support cellular multiplication.

The fact that autoclaved fermented samples allowed growth in nutrient-rich culture media but not without any nutrient supplementation also reinforces the idea of fermentation metabolites as crucial factors for the previously observed growth inhibition under longer exposure time observed when samples were directly inoculated with these microorganisms (Figure 1).

On the other hand, yeasts exhibited growth even in non-supplemented agar medium, demonstrating their greater physiological resistance and metabolic ability to utilize compounds present in the samples themselves as sources of carbon and energy, including in rosemary and eucalyptus meads (Figure 3). This behavior suggests a higher adaptability of yeasts to nutritional stress conditions, especially in fermented matrices with reduced availability of easily assimilable nutrients.

Overall, the results demonstrate that the antimicrobial activity of the evaluated beverages is strongly dependent on the matrix composition and nutrient availability of the medium. In nutrient-rich environments, antimicrobial efficacy tends to be reduced due to the promotion of metabolic recovery and microbial growth, whereas under restrictive conditions, substrate limitation enhances the inhibitory effects of the fermented matrices.

## 4. Conclusions

In conclusion, the thermally treated meads produced from eucalyptus (EM) and rosemary (RM) honey through mixed fermentation with the probiotic yeast *Saccharomyces cerevisiae* var. *boulardii* and kombucha microorganisms exhibited high antimicrobial activity against Gram-positive and Gram-negative bacteria, inhibiting the growth of *Escherichia coli*, *Salmonella Typhimurium*, *Listeria innocua*, *Staphylococcus aureus*, and *Bacillus cereus* after fermentation. The stronger activity of the thermally treated fermented matrices compared with the corresponding musts with no nutrient supplementation suggests that fermentation-related compositional changes contributed to bacterial inhibition, including the decrease in fermentable sugars and the production of ethanol and organic acids, which acted synergistically with the acidity and bioactive compounds naturally present in honey. In contrast, the meads showed limited and inefficient activity against the contaminating yeasts *Pichia manshurica*, *Candida stellata*, *Millerozyma farinosa*, *Zygosaccharomyces bailii*, and *Zygosaccharomyces rouxii*, highlighting the high tolerance of these species to the physicochemical conditions of the samples. Furthermore, in simulated food systems with high nutrient availability, no inhibitory effect was observed at matrix concentrations of up to 50%, indicating that the antimicrobial effectiveness of the meads is matrix-dependent and tends to decrease in substrate-rich environments. Therefore, these results provide preliminary evidence of in vitro antibacterial activity in autoclaved fermented matrices, although this activity was ineffective against spoilage yeasts and absent in nutrient-rich laboratory media. Importantly, the antimicrobial activity reported in this study refers specifically to heat-treated fermented matrices and does not necessarily apply to untreated fermented beverages. Further studies in real food systems are required to evaluate bactericidal activity, effective concentrations, sensory quality, safety, and probiotic functionality.

## Figures and Tables

**Table 1:** Microorganisms used for the evaluation of the antimicrobial activity of the matrices.

Group	Microorganism	Library Code
Pathogenic bacteria	*Escherichia coli*	BISA 3967
	*Salmonella enterica*	BISA 3969
	*Listeria innocua* *	BISA 3008
	*Staphylococcus aureus*	BISA 3966
	*Bacillus cereus*	BISA 4043
Contaminating yeasts	*Pichia manshurica*	ISA 2426
	*Candida stellata*	ISA 2339
	*Millerozyma farinosa*	CBS 7064
	*Zygosaccharomyces bailii*	ISA 2422
	*Zygosaccharomyces rouxii*	ISA 1770

* Nonpathogenic surrogate for *Listeria monocytogenes*.

**Table 2:** Composition of the culture media used for systems with different nutrient availabilities.

Systems	Basal Medium (% *v*/*v*)	Sample (% *v*/*v*)	Sterile Water (% *v*/*v*)
M1	50 YPD	10	40
M2	50 YPD	25	25
M3	50 YPD	50	0
M4	50 BHI	10	40
M5	50 BHI	25	25
M6	50 BHI	50	0
M7	50 Agar	10	40
M8	50 Agar	25	25
M9	50 Agar	50	0

YPD = Yeast Extract Peptone Dextrose; BHI = Brain Heart Infusion.

**Table 3:** Reducing sugar, organic acid, and volatile compound profiles of the analyzed products.

Parameters	Matrix						
	EHM	RHM	K	EM-T0	RM-T0	EM	RM
Organic Acids (g/100 g)							
Acetic	0.050 ± 0.017 ^a^	0.006 ± 0.002 ^A^	0.250 ± 0.013	0.059 ± 0.001 ^a^	0.007 ± 0.000 ^A^	0.074 ± 0.009 ^a^	0.039 ± 0.001 ^A^
Ascorbic	0.089 ± 0.003 ^a^	0.089 ± 0.000 ^A^	ND	0.091 ± 0.006 ^a^	0.086 ± 0.001 ^B^	0.085 ± 0.004 ^a^	0.100 ± 0.001 ^C^
Citric	0.687 ± 0.026 ^a^	0.029 ± 0.000 ^A^	0.094 ± 0.018	0.045 ± 0.006 ^a^	0.029 ± 0.002 ^A^	0.053 ± 0.008 ^a^	0.031 ± 0.004 ^A^
Formic	ND	0.021 ± 0.002 ^A^	0.036 ± 0.004	0.038 ± 0.002 ^b^	0.018 ± 0.000 ^B^	0.070 ± 0.001 ^a^	0.026 ± 0.003 ^C^
Lactic	ND	0.025 ± 0.008 ^A^	0.156 ± 0.012	0.042 ± 0.005 ^a^	0.019 ± 0.004 ^A^	0.039 ± 0.014 ^a^	0.046 ± 0.002 ^A^
Malic	0.130 ± 0.002 ^a^	0.079 ± 0.006 ^A^	0.243 ± 0.028	0.131 ± 0.003 ^a^	0.071 ± 0.002 ^B^	0.311 ± 0.020 ^a^	0.148 ± 0.002 ^C^
Oxalic	0.004 ± 0.000 ^a^	0.002 ± 0.000 ^A^	0.007 ± 0.000	0.004 ± 0.000 ^ab^	0.001 ± 0.000 ^A^	0.002 ± 0.001 ^b^	0.002 ± 0.000 ^A^
Tartaric	ND	0.003 ± 0.001 ^A^	ND	ND	0.003 ± 0.000 ^A^	0.010 ± 0.000	0.007 ± 0.001 ^A^
Reducing sugars (g/100 g)							
Fructose	12.221 ± 0.037 ^a^	13.415 ± 0.136 ^A^	2.599 ± 0.012	11.996 ± 0.075 ^b^	12.914 ± 0.145 ^B^	10.007 ± 0.035 ^c^	12.464 ± 0.116 ^C^
Glucose	8.578 ± 0.031 ^a^	8.705 ± 0.113 ^A^	3.036 ± 0.018	8.345 ± 0.068 ^b^	8.364 ± 0.091 ^B^	4.937 ± 0.025 ^c^	7.018 ± 0.152 ^C^
Maltose	9.099 ± 0.023 ^a^	7.757 ± 0.095 ^A^	ND	8.788 ± 0.064 ^b^	7.437 ± 0.088 ^A^	9.463 ± 0.046 ^c^	7.748 ± 0.074 ^A^
Maltotriose	0.045 ± 0.001 ^a^	0.039 ± 0.000 ^B^	ND	0.046 ± 0.000 ^a^	0.039 ± 0.000 ^AB^	0.060 ± 0.001 ^a^	0.054 ± 0.001 ^A^
Alcohols (mL/100 mL)							
Glycerol	ND	ND	ND	ND	ND	0.449 ± 0.004	0.215 ± 0.001
Ethanol (%)	ND	ND	0.030 ± 0.001	0.013 ± 0.002 ^b^	0.018 ± 0.001 ^B^	1.201 ± 0.011 ^a^	0.481 ± 0.009 ^A^

ND = Not detected concentration; EHM = Eucalyptus honey must without microorganism inoculation; RHM = Rosemary honey must without microorganism inoculation; K = Kombucha; EM-T0 = Eucalyptus honey must immediately after microorganism inoculation; RM-T0 = Rosemary honey must immediately after microorganism inoculation; EM = Fermented eucalyptus mead; RM = Fermented rosemary mead. Values are expressed as mean ± standard deviation (*n* = 3). Different letters within the same parameter indicate statistically significant differences (α = 0.05). Lowercase letters refer to comparisons among samples produced using eucalyptus blossom honey, whereas uppercase letters refer to samples produced using rosemary blossom honey.

## Data Availability

The original contributions presented in this study are included in the article. Further inquiries can be directed to the corresponding authors.
